# Biomolecular Condensates in Combined and Recurrent Plant Stresses: Integrating Phase Separation, Signal Prioritization, and Cross-Stress Memory

**DOI:** 10.3390/ijms27104520

**Published:** 2026-05-18

**Authors:** Sajid Ali, Yong-Sun Moon

**Affiliations:** Department of Horticulture and Life Science, Yeungnam University, Gyeongsan 38541, Republic of Korea

**Keywords:** biomolecular condensates, phase separation, plant stress responses, signal prioritization, stress memory, cross-stress acclimation

## Abstract

Plants frequently encounter overlapping, sequential, and recurrent stresses, but the cellular mechanisms that organize responses to these complex conditions remain incompletely understood. Biomolecular condensates are membrane-less assemblies formed through phase separation and multivalent molecular interactions, and they can regulate RNA metabolism, protein sequestration, signaling specificity, transcriptional control, and stress recovery. This review evaluates the hypothesis that plant condensates may contribute to the organization of combined and recurrent stress responses by modulating molecular accessibility, transcript fate, proteostasis, and regulatory crosstalk. We synthesize current knowledge on stress granules, processing bodies, nuclear condensates, plastid-associated condensate-like assemblies, and other stress-responsive compartments, with emphasis on their possible roles in signal filtering, RNA triage, and recovery-associated reprogramming. We also distinguish established evidence from emerging hypotheses, particularly regarding condensate-mediated signal prioritization and stress memory. Current data support condensates as rapid stress-responsive organizers, but direct evidence for their persistence after recovery or their causal roles under simultaneous multi-stress conditions remains limited. By integrating phase separation biology with plant multi-stress physiology, this review proposes a testable conceptual framework and identifies methodological priorities for future studies in plant stress resilience and crop improvement.

## 1. Introduction

Plants in natural and agricultural ecosystems are rarely challenged by a single environmental stress acting in isolation [1]. Instead, they are repeatedly exposed to complex combinations of abiotic and biotic constraints, including drought accompanied by heat, salinity coupled with oxidative imbalance, flooding followed by reoxygenation or drought, and abiotic stress superimposed on pathogen pressure [2,3]. These stress constellations are further complicated by recurrence, because the same stress or a different one may return after a short or prolonged recovery interval [4]. As a result, plant survival depends not only on rapid stress perception, but also on the capacity to distinguish among overlapping signals, allocate limited metabolic resources, and balance growth, defense, and recovery across time [5]. Recent reviews on combined and sequential stress acclimation have emphasized that plant responses under such dynamic environments are not simple additive versions of single-stress responses; instead, they involve distinct molecular states, altered signaling hierarchies, and context-dependent acclimation trajectories that remain incompletely understood [6]. This complexity creates a strong need for regulatory frameworks that can explain how plant cells integrate multiple inputs while remaining sufficiently plastic to respond to fluctuating environmental conditions [7,8].

In this review, “complex stress” refers to simultaneous, sequential, or recurrent exposure to two or more environmental constraints that produce responses distinct from those caused by a single stress alone. Simultaneous complex stress includes conditions such as heat–drought, salinity–heat, or high light–drought exposure, whereas sequential stress refers to one stress occurring before another after a partial or complete recovery interval. Recurrent stress describes repeated exposure to the same or related stress over time. Because stress thresholds vary among species, tissues, developmental stages, growth conditions, and experimental systems, complex stress should not be defined only by a fixed numerical cut-off [8,9]. For instance, heat–drought stress may be operationally defined by the co-occurrence of elevated temperature with reduced water availability, declining soil or substrate moisture, or reduced leaf water status, but the biological meaning of this combination depends on the intensity, duration, and timing of both factors. Importantly, heat–drought stress is not simply the sum of heat stress and drought stress: heat accelerates protein misfolding, transpiration, and membrane instability, whereas drought restricts water status, stomatal conductance, and carbon assimilation. Their combination can therefore generate non-additive redox, hormonal, translational, and proteostasis-related states that are not fully predictable from either stress alone. This distinction is directly relevant to biomolecular condensates, because heat-responsive assemblies such as stress granules and chaperone-enriched protein-protection condensates can form rapidly, change client composition, and undergo active remodeling during recovery, providing a useful model for understanding how complex stress reshapes condensate behavior.

In parallel with this shift toward more realistic stress biology, biomolecular condensates have emerged as an important conceptual and experimental frontier in plant molecular research [3,9]. These membrane-less compartments arise through phase separation and other multivalent assembly processes involving proteins, RNAs, intrinsically disordered regions, post-translational modifications, and changing physicochemical conditions within the cell [10,11]. Rather than serving as static storage bodies, condensates can selectively concentrate or exclude molecular components, thereby reshaping reaction environments and regulating signaling, transcription, RNA processing, translation, and protein turnover with remarkable speed and reversibility [12]. In plants, stress granules, processing bodies, nuclear condensates, and other related assemblies are increasingly recognized as stress-responsive structures that help organize cellular responses to adverse environments [13,14].

Despite this progress, most discussions of plant condensates still focus on single-stress conditions or broad descriptions of stress-responsive assemblies [15,16]. This leaves an important gap: how do condensate formation, dissolution, composition, and material properties change when plants face simultaneous, sequential, or recurrent stresses [17,18]? This question matters because combined stress does not simply activate more pathways at once; it can generate distinct redox states, hormone balances, RNA-regulatory demands, and recovery requirements [19,20]. A condensate-centered perspective may therefore help explain how stress information is physically organized, filtered, or buffered under complex environmental scenarios, while also identifying where direct experimental evidence is still missing [21].

One particularly compelling possibility is that biomolecular condensates function as hubs for signal prioritization during complex stress exposure [21,22]. Plant stress tolerance depends on tightly interconnected networks involving abscisic acid, salicylic acid, jasmonates, ethylene, calcium fluxes, reactive oxygen species, redox cues, and diverse RNA-centered regulatory pathways, all of which can interact cooperatively or antagonistically depending on stress identity and order [23,24]. Condensates are well-suited to participate in this integration because they can respond rapidly to changes in molecular concentration, phosphorylation status, ionic conditions, osmotic stress, temperature, and RNA abundance, while also reorganizing which transcripts or proteins remain accessible for translation, sequestration, or decay [21]. In principle, this allows condensates to act as dynamic biochemical filters that help determine which cellular programs are temporarily paused, which are amplified, and which are buffered during acute stress combinations [25]. Such a framework is especially attractive for plant stress biology because it offers a mechanistic basis for understanding why identical signaling components may produce different outcomes under different stress contexts, intensities, or temporal sequences [26].

A second underexplored dimension is the possible relationship between condensates, stress memory, and cross-stress acclimation [27]. Plants can retain information from prior stress exposure through physiological, transcriptional, chromatin-based, and RNA-mediated mechanisms, allowing them to respond differently when the same or a related stress is encountered again [28]. However, direct evidence that plant condensates physically persist after stress recovery and thereby store memory remains limited. Current evidence more strongly supports roles for condensates in rapid sensing, RNA regulation, proteostasis, and immediate stress-response organization. Therefore, in this review, condensates are not treated as established memory modules. Instead, we consider whether stress-induced changes in condensate composition, material properties, dissolution kinetics, or reassembly thresholds could contribute indirectly to priming, recall, or cross-stress acclimation. This hypothesis provides a testable bridge between phase separation biology and plant stress-memory research, but it requires direct recovery-phase experiments.

Against this background, the present review examines the hypothesis that biomolecular condensates may serve as active organizers of plant responses to combined and recurrent stresses rather than only as passive by-products of cellular perturbation [29]. We first summarize the principles, classes, and functional properties of stress-associated condensates in plant cells, and then place them within the biologically realistic context of co-occurring, sequential, and repeated stresses. Building on current knowledge of hormone signaling, ROS and calcium networks, RNA metabolism, translation control, transcriptional reprogramming, and stress memory, we argue that condensates may represent an overlooked mechanistic interface between rapid stress perception and longer-term acclimatory behavior. By focusing on phase separation, signal prioritization, and cross-stress memory, this review aims to provide a coherent conceptual framework for understanding how plants manage environmental complexity and to identify the most important experimental and translational questions for future work in stress-resilient crop biology [30]. This emphasis is well aligned with the current interest in molecular mechanisms of plant stress tolerance, including signaling, transcriptional regulation, and adaptive plasticity under diverse biotic and abiotic challenges.

## 2. Biomolecular Condensates in Plant Cells: Concepts, Properties, and Stress-Relevant Types

Biomolecular condensates have emerged as an important framework for understanding how plant cells achieve rapid and reversible intracellular organization without relying on surrounding membranes [31]. In contrast to membrane-bound organelles, these assemblies arise through collective interactions among proteins, RNAs, metabolites, and other macromolecules, allowing cells to concentrate selected molecules while excluding others within dynamic microenvironments [32]. In plants, this mode of organization is increasingly linked to stress acclimation because condensates can be induced or remodeled by environmental and developmental cues, and in turn influence RNA metabolism, signaling, translation, transcription, and other processes that must be rapidly reprogrammed during stress [17]. Current plant literature, therefore, treats condensates not simply as unusual cellular bodies but as functional organizers of biochemical reactions whose composition, dynamics, and material properties are directly relevant to stress tolerance.

### 2.1. Molecular Principles of Biomolecular Condensate Formation

Most biomolecular condensates are thought to form through phase separation and related multivalent assembly processes, although the term “biomolecular condensate” is often preferred because it accommodates a wider range of material states and mechanisms than a simple liquid–liquid model [33]. Their assembly is usually driven by weak but cooperative interactions among intrinsically disordered regions, low-complexity sequences, folded multimerization domains, RNA molecules, and scaffold–client relationships that together enable selective enrichment of macromolecules in dense phases [34]. In plants, condensate formation is highly sensitive to intracellular context, including temperature, redox state, pH, ionic strength, osmotic conditions, and protein concentration, which makes this organizational mode particularly suitable for environmental sensing [14]. Recent reviews also emphasize that condensates should not be viewed as uniform liquid droplets; rather, they can display viscoelastic, gel-like, or age-dependent properties, and these material states can strongly influence molecular residence times, exchange kinetics, and ultimately biological function [35,36]. Post-translational modifications further modulate this behavior by altering interaction valency, charge distribution, and assembly thresholds, thereby tuning condensate formation or dissolution during stress responses [37].

### 2.2. Major Classes of Plant Biomolecular Condensates Associated with Stress Responses

Among the best-characterized stress-associated condensates in plants are stress granules (SGs) and processing bodies (PBs)**,** both of which are central to post-transcriptional regulation [38]. SGs are typically stress-induced assemblies that accumulate untranslated mRNAs together with RNA-binding proteins, translation factors, chaperones, ATPases, and additional regulatory molecules when cells experience adverse conditions [39]. PBs, in contrast, are often present constitutively but can be remodeled by stress and are classically associated with mRNA decapping, turnover, storage, and translational repression. Rather than functioning as isolated compartments, SGs and PBs appear to participate in a dynamic continuum of RNA fate control in which transcripts can be stored, degraded, protected, or redirected depending on cellular need [40]. Recent plant studies also indicate that these condensates contain not only proteins and mRNAs but also long non-coding RNAs and metabolites, reinforcing the idea that they are chemically and functionally complex assemblies rather than simple RNA aggregates [41].

**Figure 1:**
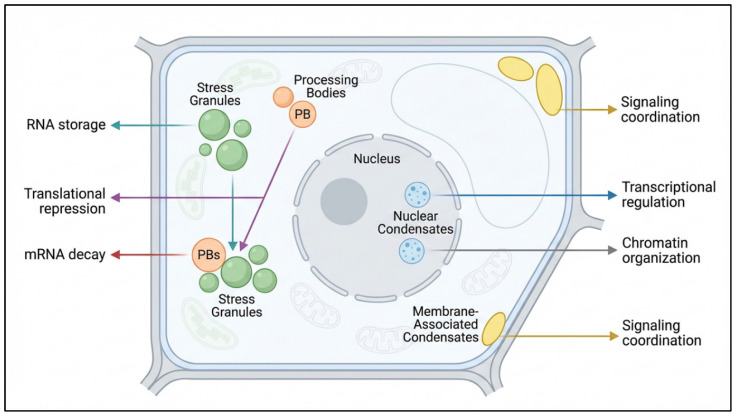
Major classes of biomolecular condensates in plant cells and their roles in stress responses. Schematic representation of major membrane-less compartments in plant cells, including stress granules (SGs), processing bodies (PBs), nuclear condensates, membrane-associated condensates, and plastid/chloroplast-associated stress granule-like assemblies. Stress granules are associated with mRNA storage and translational repression, whereas processing bodies contribute to mRNA decay and RNA turnover. Nuclear condensates are linked to transcriptional regulation and chromatin organization, while membrane-associated condensates participate in signaling coordination. Plastid- or chloroplast-associated condensate-like assemblies may connect photosynthetic stress, redox signaling, organelle RNA/protein protection, and plastid-to-nucleus communication with broader cellular stress responses. Together, these dynamic assemblies help spatially organize gene expression, organelle communication, and signaling during plant stress responses. Created by the authors using FigureLab based on concepts synthesized from the literature.

Beyond SGs and PBs, plant cells contain a broader and increasingly recognized spectrum of condensates distributed across the nucleus, cytoplasm, membrane interfaces, and other subcellular locations [42]. A schematic overview of the major plant biomolecular condensates and their stress-related functions is shown in Figure 1. In recent years, studies have highlighted nuclear condensates linked to transcriptional regulation, chromatin organization, and gene-expression control, along with condensates positioned at or influenced by membranes, where phase behavior may intersect with signaling, trafficking, and environmental responsiveness [43,44]. From the perspective of stress biology, this diversity is important because different condensate classes likely regulate different layers of cellular adaptation: some reorganize RNA metabolism in the cytoplasm, some shape transcriptional or epigenetic outputs in the nucleus, and others may couple phase behavior to membrane-associated signaling or organelle communication [45]. Thus, the stress relevance of plant condensates lies not only in the existence of SGs and PBs, but in the broader possibility that multiple membrane-less assemblies collectively partition and coordinate responses across cellular compartments.

A particularly plant-specific dimension of condensate biology is the relationship between stress-responsive condensates and chloroplast-centered stress physiology. Unlike animal cells, plant cells must coordinate cytoplasmic stress responses with photosynthesis, plastid redox balance, retrograde signaling, and light-dependent metabolic regulation. Heat, drought, salinity, and high-light stress can disturb chloroplast protein folding, photosynthetic electron transport, and reactive oxygen species production, thereby generating signals that influence cytoplasmic RNA metabolism and stress-granule behavior. Recent evidence for plastidial or chloroplast-associated stress granule-like structures suggests that condensate biology in plants extends beyond classical cytoplasmic SGs and PBs. These plastid-related assemblies may help protect or reorganize chloroplast-localized RNAs and proteins during stress, whereas cytoplasmic SGs may indirectly respond to chloroplast-derived redox and metabolic cues. Therefore, interactions among stress granules, chloroplast stress signals, and plastid-associated condensates represent an important plant-specific layer of condensate-mediated stress regulation. This perspective strengthens the view that plant condensates should be interpreted not only as general eukaryotic membrane-less compartments, but also as structures integrated with photosynthetic organelles, plastid-to-nucleus signaling, and plant-specific acclimation programs.

### 2.3. Functional Properties of Condensates in Cellular Regulation

The biological importance of biomolecular condensates lies in their ability to create selective and tunable biochemical environments. By concentrating particular proteins, RNAs, metabolites, or signaling factors while excluding others, condensates can accelerate, suppress, buffer, or spatially redirect biochemical reactions [46,47]. In plant systems, this has major implications for stress regulation because condensates can influence transcript triage, translational repression, mRNA turnover, transcriptional control, and the local availability of regulatory proteins during periods of rapid environmental change [48]. Their reversible nature is especially relevant: condensates can assemble quickly when stress is encountered and dissolve or remodel during recovery, allowing cells to shift between protective arrest, active response, and re-entry into growth [49]. At the same time, their material properties are not fixed, and age-dependent transitions or altered exchange dynamics may affect how long specific regulators are retained, how efficiently molecules move across condensate boundaries, and whether an assembly remains adaptive or becomes less functional [18]. These features make condensates particularly attractive candidates for regulating stress signal integration, response prioritization, and possibly the persistence of molecular states relevant to stress memory.

## 3. Combined and Recurrent Plant Stresses: A New Context for Molecular Regulation

A central limitation of classical plant stress biology is that it has often relied on single-stress experimental designs, even though plants in field environments more commonly encounter stress combinations that are simultaneous, sequential, or repeatedly recurring over time [18,50]. Recent work has shown that co-occurring and sequential stresses generate physiological, developmental, and molecular outcomes that cannot be reliably inferred from single-stress datasets alone [51,52]. As a result, understanding plant stress tolerance now requires a framework that accounts not only for stress identity, but also for stress timing, overlap, recovery, and re-exposure. This shift is essential for any mechanistic model that aims to explain how intracellular regulatory systems, including biomolecular condensates, might integrate environmental complexity rather than simply respond to isolated perturbations [53].

### 3.1. Defining Combined, Sequential, and Recurrent Stresses in Plants

Combined stress generally refers to the simultaneous occurrence of two or more stresses, such as heat plus drought or salinity plus high light. In contrast, sequential stress involves one stress followed by another, with or without partial recovery between them [20]. Recurrent stress is conceptually distinct because it focuses on repeated exposure to the same stress, or sometimes a related second stress, after an intervening recovery phase [54]. These distinctions are not merely semantic; they reflect biologically different contexts of perception, signaling, and response. In simultaneous stress, plants must process multiple inputs at once, whereas in sequential stress, the first exposure can reshape the molecular landscape in which the second stress is perceived [55,56]. In recurrent stress, the key question is whether prior exposure leaves a measurable trace that modifies later output, producing priming, acclimation, or memory. Recent reviews therefore emphasize that defining stress structure precisely is necessary for interpreting plant phenotypes and molecular regulation under realistic environmental conditions [57,58].

From the perspective of stress memory, recurrent stress is especially informative because it introduces the concepts of priming, triggering, and retention of a primed state [54]. In the current conceptual framework, an initial stimulus can alter the response to a later triggering stimulus, and memory is inferred when primed and non-primed plants display quantitatively or qualitatively different outputs after that second exposure [59]. Importantly, the priming and triggering events do not need to be identical in nature or intensity, which opens the door to cross-stress effects in which one environmental challenge modifies the response to another [60]. This temporal architecture makes recurrent stress an ideal context for exploring how molecular systems preserve information over time, and it is highly relevant for the present review because condensate assembly and disassembly may themselves be influenced by stress history rather than by stress intensity alone.

### 3.2. Signal Conflict, Crosstalk, and Prioritization Under Multi-Stress Conditions

One of the most important insights from recent studies is that plant responses to combined and sequential stresses are often non-additive [55]. Experimental work in Arabidopsis using sublethal single, combined, and sequential stresses has shown that simultaneous or ordered stress exposure can produce unique phenotypic and molecular signatures that do not appear under the corresponding individual stresses [20,61]. This means that plants are not simply stacking one response on top of another; instead, they are entering distinct regulatory states shaped by signal interference, metabolic constraints, and context-specific response hierarchies [20]. Reviews on multiple abiotic stress acclimation similarly conclude that combined-stress responses involve specific signaling and regulatory outcomes that must be studied in their own right rather than inferred indirectly from single-stress models [62].

**Figure 2:**
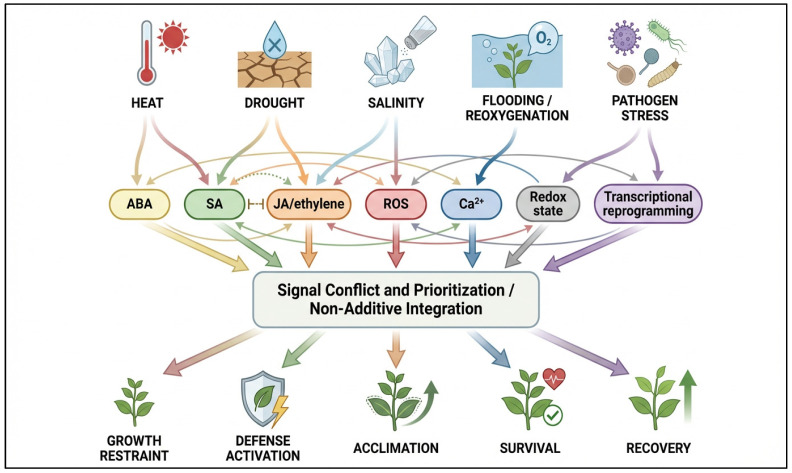
Non-additive integration of signaling pathways under combined and recurrent plant stresses. Distinct environmental stresses generate partially overlapping signaling networks involving phytohormones, ROS, Ca^2+^ fluxes, redox state, and transcriptional reprogramming. Extensive crosstalk among these pathways produces context-dependent signal conflict and prioritization rather than additive outputs. This integration determines the balance between growth restraint, defense activation, acclimation, survival, and recovery under complex stress environments. Created by the authors using FigureLab.

This non-additivity is closely tied to signal conflict and prioritization. Under complex stress conditions, plants must coordinate hormone pathways, redox changes, calcium signaling, transcriptional programs, and metabolic reallocation while also balancing growth, defense, and survival [63]. The order and coexistence of stresses can shift the dominance of one pathway over another, alter sensitivity thresholds, and redirect energy use toward immediate protection or later recovery. Because these decisions occur in a crowded intracellular environment where molecular availability and reaction space are limited, multi-stress biology increasingly points toward the need for flexible organizational systems that can filter, buffer, or triage competing signals [25,64]. This is precisely why a condensate-centered perspective becomes attractive: it offers a plausible cellular mechanism through which plants could selectively reorganize signaling and RNA/protein behavior under stress combinations rather than treating all inputs equally [44]. The integration of multiple stress-derived signals and their non-additive outcomes is summarized in Figure 2.

### 3.3. Recovery, Re-Exposure, and the Temporal Dimension of Plant Stress Adaptation

Recovery is not simply the interval between two stress events; it is an active biological phase during which the plant may reset, partially retain, or remodel the molecular consequences of prior exposure [65]. The duration of this interval strongly influences whether the primed state is maintained and whether the second stress elicits a faster, stronger, weaker, or qualitatively different response [66]. Reviews on plant stress memory emphasize that acclimation to intermittent stress depends on information retained from the first exposure, and that the persistence of this information determines the extent to which the triggering stress is modified [56]. In dehydration-memory studies, for example, repeated dehydration–rehydration cycles have been associated with enhanced expression of canonical drought-responsive genes, demonstrating that plant responses can be shaped by prior experience over several days [67].

The temporal dimension is also evident in transcriptional memory, where heat stress has become a particularly informative model for understanding how plants sustain altered responsiveness after an earlier exposure [65]. Recent work highlights chromatin-linked and transcriptional co-regulator mechanisms that can preserve a modified response state beyond the initial stress itself [68]. More broadly, this literature shows that the biological meaning of a stress event cannot be separated from its timing, recurrence, and recovery context. For the purposes of this review, that point is crucial: if condensates are to be considered candidates for signal prioritization or cross-stress memory, they must be interpreted not only as structures induced during acute stress, but also as dynamic assemblies whose persistence, composition, and reassembly thresholds may change across cycles of stress and recovery.

## 4. Condensates in Stress Signaling

Biomolecular condensates are increasingly viewed as active components of plant stress signaling rather than passive by-products of cellular perturbation. Current plant literature shows that stress-associated condensates influence several core regulatory layers, especially RNA metabolism, translational control, transcriptional regulation, and signaling organization, while their assembly is itself shaped by environmental cues [25]. This makes them particularly relevant to stress biology, because they can rapidly reorganize molecular interactions without requiring new membrane formation and can therefore respond on the same timescale as acute stress signaling. In this sense, condensates provide a plausible mechanistic bridge between early stress perception and downstream reprogramming of gene expression and metabolism.

### 4.1. Hormones, ROS, and Ca^2+^ Networks

A major strength of the condensate framework is that it fits naturally with the dynamic chemistry of plant stress signaling. Condensate assembly is sensitive to changes in temperature, redox state, pH, and molecular concentration, all of which are rapidly altered during stress exposure [69]. The Plant Cell review on stress-related condensates notes that phase separation can be promoted by shifts in the intracellular environment, including temperature and redox conditions, while salicylic acid has already been linked to the condensation of defense regulators such as NPR1- and GBPL-associated assemblies [70,71]. In addition, hypoxia-induced condensate datasets include CALMODULIN-LIKE PROTEIN 38, suggesting that calcium-linked signaling components can also intersect with stress-associated condensates [70]. Taken together, the strongest current evidence supports a model in which condensates act as responsive hubs embedded within hormone and redox signaling, while the precise mechanistic role of Ca^2+^-dependent regulation remains promising but less fully resolved [72].

### 4.2. RNA Control and Translation

The most established signaling-related role of plant condensates lies in post-transcriptional regulation. Stress granules and processing bodies are central to the control of mRNA fate, because they help determine whether transcripts are stored, decapped, degraded, translationally repressed, or returned to active translation [73,74]. The Plant Cell review emphasizes that sequestration of RNA-binding proteins and RNAs within condensates can alter the translational landscape in ways that favor survival and acclimation, while recent reviews on plant translation stress that translational regulation is now recognized as a multilayered and highly dynamic component of both abiotic and biotic stress responses [75]. Updated work on plant P-bodies further shows that these condensates are involved in mRNA decapping, degradation, translational repression, and storage under ethylene and abiotic stress conditions [76,77]. For this review, this subsection is especially important because it places condensates at the heart of selective transcript triage, which is likely to become even more critical when plants face combined or recurrent stresses that demand rapid prioritization among competing gene-expression programs. In plants, such transcript triage may also be influenced by chloroplast-derived redox and metabolic signals, suggesting that cytoplasmic SG/PB dynamics should be considered together with plastid stress signaling and organelle-specific RNA regulation.

### 4.3. Transcription and Chromatin

Condensate biology is also becoming increasingly relevant to transcriptional and chromatin-level regulation in plants [78]. Recent reviews specifically highlight emerging roles of phase separation in plant transcriptional activity, RNA-mediated chromatin silencing, and chromatin compartmentalization, indicating that condensates may influence not only cytoplasmic RNA control but also nuclear gene-regulatory architecture [79]. Broader work on transcriptional condensates likewise supports the idea that condensate-like assemblies can create selective microenvironments for transcription factors, co-regulators, and chromatin-associated proteins [80]. Importantly, new plant evidence is moving beyond theory: a 2025 Developmental Cell study reported that heat shock disrupts nuclear lamina phase separation, releasing factors for chromatin access and stress-responsive transcriptional reprogramming [81]. This makes nuclear condensates particularly relevant to the present review, because they offer a direct route by which environmental signals could be translated into persistent or state-dependent changes in gene expression, potentially linking acute signaling to stress memory. Key physicochemical and regulatory factors governing biomolecular condensate formation and dynamics are summarized in Table 1.

## 5. Condensates Under Complex Stress

Although biomolecular condensates are now well established as components of plant stress responses, most mechanistic evidence still comes from single-stress systems such as heat, hypoxia, salinity, or pathogen challenge examined separately [95]. Recent reviews on plant condensates and on multi-stress acclimation both point to the same gap: plants in nature experience simultaneous, sequential, and sometimes multifactorial stresses, yet we still know relatively little about how condensate behavior changes when multiple signals coexist or recur over time [96]. This gap is important because combined stresses often generate unique physiological and transcriptomic states that do not appear under the corresponding single stresses, implying that condensate dynamics under complex environments are unlikely to be simple extensions of single-stress behavior [97].

### 5.1. Shared and Stress-Specific Responses

A useful starting point is to distinguish between shared condensate responses and stress-specific condensate responses. Some condensates, especially stress granules and processing bodies, appear to represent broadly conserved protective strategies that help cells reorganize RNA metabolism, translation, and protein homeostasis under diverse adverse conditions [98]. Reviews of plant condensates consistently emphasize these assemblies as central stress-responsive compartments, and updated P-body literature shows that their abundance and composition change under drought, salinity, heat, and cold [99]. At the same time, recent work also indicates that particular stresses recruit distinct molecular clients or impose different physicochemical conditions, meaning that condensates formed during one stress may not be compositionally or functionally identical to those formed during another [100].

**Figure 3:**
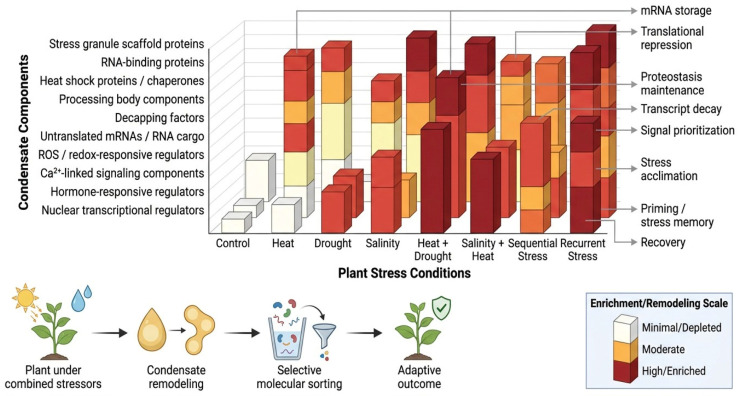
Conceptual heatmap of condensate component remodeling under different plant stress combinations. Schematic overview showing the relative enrichment, remodeling, or functional prominence of major biomolecular condensate component classes under representative plant stress contexts, including single stresses, combined stresses, sequential stresses, and recurrent stresses. Condensate component classes may include RNA-binding proteins, untranslated mRNAs, translation factors, mRNA decay factors, chaperones/heat shock proteins, proteostasis-related factors, redox-associated regulators, signaling proteins, and nuclear or plastid-associated regulators. Color intensity indicates the relative extent of recruitment or remodeling inferred from the current literature and should be interpreted as a conceptual synthesis rather than a direct quantitative dataset. Functional annotation arrows indicate the major biological outputs linked to these component changes, including mRNA storage, translational repression, RNA decay, proteostasis, signal prioritization, and stress memory/cross-stress acclimation. Created by the authors using FigureLab based on concepts synthesized from the literature.

For the present review, this distinction is especially important because combined stress may involve both layers simultaneously. A plant exposed to heat plus drought may deploy a common protective condensate framework while also generating stress-specific condensate features shaped by hormone balance, redox state, ion fluxes, or transcript availability unique to that stress combination [14]. Since multi-stress studies now show that simultaneous or ordered stresses produce unique molecular signatures rather than additive outputs, it is reasonable to propose that condensates under complex environments also acquire “hybrid” properties that cannot be predicted from single-stress observations alone [101]. At present, this remains more a conceptual model than a fully resolved mechanism, but it is strongly motivated by the broader multi-stress literature. To synthesize this concept more clearly, a heatmap-style overview of major condensate component classes across representative single, combined, sequential, and recurrent stress contexts is presented in Figure 3. The figure also highlights the major functional outputs associated with these component shifts, including RNA triage, translational repression, proteostasis, signaling integration, and stress memory-related acclimation.

This synthesis emphasizes that condensate remodeling under complex stress likely reflects both a conserved core response and stress-specific client recruitment, thereby supporting the idea that different stress combinations may generate distinct condensate states with different functional consequences.

### 5.2. Signal Prioritization

A more cautious and mechanistically explicit interpretation is that condensates may contribute to signal prioritization when plants face conflicting environmental inputs, but this function remains a working hypothesis rather than a broadly demonstrated mechanism. In this context, “prioritization” does not mean that condensates make autonomous decisions; rather, it refers to measurable changes in molecular accessibility, localization, residence time, and fate. For example, condensates may prioritize one response over another by selectively sequestering untranslated mRNAs, concentrating RNA-binding proteins, retaining or releasing signaling regulators, altering protein interaction networks, or shifting transcripts between storage, translation, and decay. These processes could influence which stress-response programs are amplified, delayed, buffered, or suppressed under combined stress. However, direct examples showing condensate formation and functional remodeling under two or more simultaneous stresses remain limited. Therefore, the present review treats condensate-mediated signal prioritization as a plausible model that is supported by known condensate properties and multi-stress non-additivity, but still requires direct validation under defined combined-stress regimes [41,102,103].

**Figure 4:**
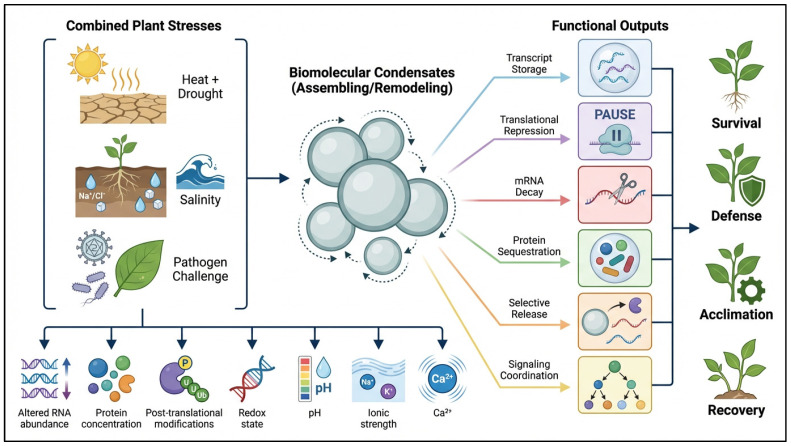
Biomolecular condensates as dynamic hubs for signal prioritization under combined plant stresses. This schematic illustrates a hypothetical framework in which combined stresses reshape the intracellular physicochemical and regulatory environment, thereby promoting condensate assembly or remodeling. By selectively storing, repressing, degrading, sequestering, or releasing RNAs and proteins, biomolecular condensates may function as context-dependent filters that coordinate downstream programs linked to survival, defense, acclimation, and recovery. Created by the authors using FigureLab.

This framework is especially powerful under combined stress, where signal competition is unavoidable [104]. Multi-stress acclimation reviews show that co-occurring and sequential stresses reshape signaling hierarchies and growth, defense tradeoffs, while current abiotic-stress syntheses emphasize that these responses are interconnected through shared hormonal, redox, and transcriptional networks [96]. Within such crowded regulatory space, condensates could provide one physical mechanism by which selected RNAs or proteins are sequestered, stabilized, released, excluded, or redirected toward translation or decay, thereby contributing to rapid redistribution of cellular resources [105]. Operationally, this hypothesis should be tested by comparing condensate number, size, composition, exchange dynamics, and RNA/protein cargo under single stress, simultaneous stress combinations, sequential stress, and recovery. The evidence is still incomplete, but the model fits both the known properties of condensates and the known non-additivity of plant multi-stress responses. A proposed model for condensate-mediated signal prioritization under complex stress is presented in Figure 4.

### 5.3. Sequence and Recurrence

The order, intensity, and recurrence of stress exposures are likely to be major determinants of condensate behavior [106]. Recent studies and reviews on sequential stress acclimation emphasize that heat followed by drought is not biologically equivalent to drought followed by heat, because the first stress can alter the molecular state in which the second stress is perceived [107,108]. Likewise, recurrent stress introduces a memory phase in which prior exposure may influence the threshold, speed, or composition of a later response. From a condensate perspective, this means that the same assembly might form more rapidly, recruit different clients, persist longer, or dissolve differently after repeated exposure compared with a first encounter [109,110].

This temporal dimension is where condensate biology becomes particularly interesting for plant stress theory. General condensate reviews increasingly describe these assemblies as dynamic sensors and regulators rather than passive structures, and emerging work on condensates as cellular memory modules proposes that their assembly history can shape later behavior [14]. When combined with current plant stress-memory frameworks, this supports the idea that recurrence may modify condensate “set points,” including nucleation thresholds, residence times, or recovery kinetics [110]. Even where direct plant evidence remains limited, this line of reasoning provides a coherent mechanistic bridge between phase separation and the well-established observation that prior stress exposure changes later plant responses [111].

## 6. Condensates and Stress Memory

Stress memory has become a major theme in plant biology because repeated or prior stress exposure often changes the magnitude, speed, or quality of later responses [4]. Current reviews describe plant stress memory as involving priming, retention during a recovery phase, and altered output after a later triggering stress, with contributions from physiological, transcriptional, chromatin-based, and RNA-centered mechanisms. However, the subcellular structures that might help organize or preserve these altered states are still not fully understood. This is precisely where condensate biology becomes relevant, because membrane-less assemblies are inherently dynamic, stimulus-responsive, and capable of reorganizing molecular interactions in ways that could extend beyond the original stress event.

### 6.1. Priming and Recall

A key concept in stress memory is that the first exposure does not merely trigger an immediate defense response; it can also leave the cell in a modified state that changes how a later stress is perceived [4]. In plants, this is often described in terms of priming and recall, where the initial stress establishes a memory phase and the subsequent stress elicits a different response compared with a non-primed plant [112]. Reviews on abiotic stress memory emphasize that such effects are strongly dependent on the duration of the recovery interval, the type of priming stimulus, and the identity of the later trigger [113]. These principles are compatible with condensate biology, because condensates can assemble rapidly and remodel continuously; however, direct evidence that plant stress condensates persist through recovery as physical memory carriers remains limited [114].

The most defensible argument at present is not that condensates have been proven to be plant memory devices, but that they may influence memory-related responses indirectly by changing the biochemical state of the cell during and after stress [109,110]. For example, an initial stress could alter condensate composition, client selection, material properties, dissolution kinetics, or later nucleation thresholds. Such changes might allow a second stress to trigger faster or more selective RNA regulation, protein sequestration, or signaling reorganization even after visible recovery. However, this remains a hypothesis because direct demonstrations of condensate persistence or recovery-phase condensate tracking in plants are still scarce. Therefore, stress memory should continue to be interpreted primarily through established physiological, transcriptional, chromatin-based, and RNA-mediated mechanisms, with condensates considered as possible organizing interfaces that require further validation [115]. Different forms of stress memory and their potential links to biomolecular condensate dynamics are summarized in Table 2.

### 6.2. Links to Chromatin and RNAs

The strongest routes by which condensates could contribute to plant stress memory are likely through RNA-centered regulation and nuclear/chromatin-associated regulation [124]. On the RNA side, stress granules and P-bodies already provide a clear framework for selective transcript storage, decay, and translational repression, all of which can influence whether stress-associated transcripts remain available for rapid reuse [125]. On the nuclear side, recent plant reviews point to emerging roles of condensates in transcriptional control, RNA-mediated chromatin silencing, and chromatin compartmentalization, while newer work shows that stress can directly affect nuclear phase behavior and chromatin accessibility [126]. Together, these findings suggest that condensates could interface with established memory pathways by shaping both cytoplasmic transcript fate and nuclear transcriptional competence [127].

This connection is important because plant stress memory is already known to involve chromatin remodeling, transcriptional reprogramming, and RNA-based regulation [128]. Recent reviews of stress memory and epigenetic memory networks underscore that altered chromatin states and regulatory RNAs are central to the persistence of acclimatory information [129]. Condensates do not replace these mechanisms; rather, they may help organize them spatially and temporally. In that sense, condensates may act as intermediates between rapid stress perception and more durable regulatory change, thereby linking immediate response architecture to longer-lasting memory states [31,130].

### 6.3. Cross-Stress Memory

Cross-stress memory is particularly relevant to the theme of this review because it asks whether exposure to one stress can alter the response to a later, different stress [131,132]. Current memory frameworks in plant biology allow for this possibility, and reviews on recurrent and combined stress acclimation increasingly treat cross-stress effects as a realistic outcome of environmental history rather than a rare exception [113,133]. This matters because plants in field settings often experience stress sequences rather than identical repeated events. A prior episode of heat, drought, flooding, or oxidative stress may therefore reshape later responses to a distinct challenge by altering shared signaling hubs, metabolic state, or transcriptional readiness [134].

**Figure 5:**
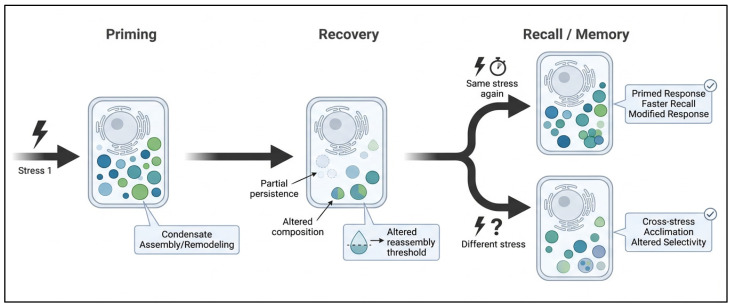
Hypothesis-testing model for condensate involvement in plant stress memory and cross-stress acclimation. The revised model distinguishes established stress-memory processes from hypothesized condensate-linked mechanisms. During the first stress exposure, condensates may assemble or remodel rapidly and contribute to RNA triage, protein sequestration, proteostasis, or signaling reorganization. During recovery, established memory mechanisms include transcriptional, chromatin-based, physiological, and RNA-mediated changes, whereas condensate persistence or altered reassembly thresholds remain hypothetical and require direct experimental validation. Upon recurrent or different secondary stress exposure, altered condensate nucleation, client recruitment, dissolution kinetics, or RNA/protein accessibility may contribute to priming or cross-stress acclimation. Solid arrows indicate established stress-memory pathways, and dashed arrows indicate proposed condensate-mediated mechanisms that remain to be tested. Created by the authors using FigureLab.

Here again, condensates provide an attractive but underexplored mechanism. Because many condensates respond to shared variables such as RNA abundance, protein concentration, redox status, and post-translational modification, a condensate altered by one stress could plausibly influence how the cell handles another [125]. This would be especially relevant for assemblies that regulate common signaling nodes or transcript pools. Direct demonstrations in plants remain limited, so this should be presented cautiously, but as a review concept it is both timely and original: condensates may be part of the molecular infrastructure through which one stress experience is translated into altered preparedness for another [42]. A hypothetical framework linking biomolecular condensate dynamics to stress priming, recall, and cross-stress memory is illustrated in Figure 5.

## 7. Approaches, Gaps, and Translation

The promise of condensate biology in plant stress research is clear, but the field still faces major conceptual, experimental, imaging, and translational limitations. Recent best-practice papers on condensate research emphasize that condensates cannot be defined by appearance alone; their composition, reversibility, material properties, and functional consequences must all be rigorously tested [135,136]. At the same time, multi-stress plant biology is itself methodologically challenging, because simultaneous and sequential stresses require careful control of intensity, duration, and recovery. For this reason, progress in understanding condensate-mediated stress adaptation will depend not only on new hypotheses, but also on better experimental design that brings condensate methods and realistic stress biology together [55,137]. A major priority is to move beyond abrupt single-stress assays toward experimental systems that capture simultaneous, sequential, and recurrent stress regimes more realistically [55]. Recent Arabidopsis work using sublethal combined and sequential stresses demonstrates how strongly phenotype and transcriptome can depend on stress structure, while broader multi-stress reviews argue that realistic combinations are essential for understanding plant resilience in dynamic environments [96]. For condensate studies, this means that imaging or proteomic analysis performed under a single acute stress may reveal only part of the biology. Future work should therefore incorporate stress order, recovery windows, and re-exposure, and should explicitly test whether condensate assembly thresholds, dissolution kinetics, material properties, and client composition differ across these contexts [138]. Particular attention should be given to the dynamic component composition of condensates in response to stressors of different physical and biochemical natures, because changes in scaffold proteins, client proteins, RNAs, chaperones, signaling factors, and post-translational modifications may provide the most direct route to understanding condensate function.

Methodologically, the field already has a strong toolkit, including live-cell imaging, fluorescence recovery approaches, super-resolution microscopy, in vitro reconstitution, genetics, proteomics, crosslinking-mass spectrometry, single-cell transcriptomics, and emerging synthetic systems [139,140]. However, these approaches are most useful when matched to clearly defined biological problems rather than applied as isolated descriptive tools. For example, live-cell and super-resolution imaging are suited to monitoring condensate nucleation, fusion, dissolution, and recovery dynamics; fluorescence recovery after photobleaching can quantify molecular exchange and material properties; crosslinking-mass spectrometry can help capture weak or transient protein–protein and protein–RNA interaction networks; and single-cell or spatial transcriptomics can reveal cell-type-specific heterogeneity in condensate-associated stress responses. Current practice papers stress the need to combine morphology-based observations with quantitative biophysics, interaction mapping, functional perturbation, and stress-phenotype validation, while recent synthetic condensate work shows that engineered plant protein systems can help dissect phase behavior experimentally [141]. In the context of this review, such tools are especially valuable because they can distinguish whether a stress-induced body is a functional regulatory condensate, a transient protective assembly, or merely a by-product of severe perturbation [84,142].

To clarify how these methods can be selected according to specific experimental and translational needs, a technology–problem matching framework is provided in Table 3. Several unresolved questions should define the next phase of the field. First, we still do not know how many plant stress condensates are truly causal for tolerance rather than correlative markers of stress [143]. Second, it remains unclear whether different stress combinations generate distinct “condensate codes” in terms of composition, material state, or subcellular distribution [99]. Third, the persistence required for condensate-based memory is not yet established: must a condensate physically persist through recovery, or is it enough that prior stress changes its later reassembly behavior? Recent reviews in plant condensates, plant immunity, and plant stress memory all converge on the need for this kind of mechanistic clarification.

As summarized in Table 3, imaging-based and sensor-based approaches are central to condensate research, but plant condensate imaging also faces technical barriers that are less prominent in many animal or microbial systems. The cell wall can reduce imaging depth, limit probe delivery, increase light scattering, and complicate high-resolution observation of small or fast-moving condensates. Large vacuoles, chlorophyll autofluorescence, tissue thickness, phototoxicity, and stress artifacts caused by sample mounting can further interfere with live imaging, especially when heat, drought, salinity, or high-light treatments are applied during microscopy. These limitations can be addressed by combining optimized optical clearing or gentle mounting systems, low-phototoxicity imaging, genetically encoded fluorescent reporters, light-sheet or spinning-disk microscopy, super-resolution approaches, and quantitative image analysis. In addition, nanosensors and field-compatible biosensors offer a complementary solution by allowing real-time detection of stress-linked variables such as ROS, Ca^2+^, pH, hormones, metabolites, or nutrient status in living tissues. Such sensors do not replace condensate imaging, but they help connect condensate behavior with measurable physiological stress signals and agriculturally relevant outcomes.

Another gap concerns scale. Most studies still focus on one condensate type, one tissue, and one stress, whereas future research will need to connect condensates across compartments, cell types, and time [144]. The growing literature on multifactorial stress combinations and panomics-based management of combined stress suggests that high-dimensional approaches will become increasingly important [145]. That trend fits well with the present review, because condensates are unlikely to function as isolated modules; they probably operate within broader molecular networks whose structure changes with environmental history [110]. Although this field is still mechanistically young, its translational potential should be discussed cautiously because much of the direct mechanistic evidence for plant condensates still comes from Arabidopsis and other model systems. This model-to-crop gap is a major limitation for agricultural translation. Several crop studies have shown that combined, sequential, or recurrent stresses generate non-additive physiological, transcriptomic, hormonal, or memory-related responses in species such as soybean, cotton, Brassica, creeping bentgrass, and other crop systems [50,53,54,57,58,66]. However, these studies generally do not yet resolve whether condensates directly mediate such responses. Conversely, many mechanistic studies of condensate formation, stress granules, processing bodies, and phase-separating regulators have been performed in Arabidopsis or simplified experimental systems. Therefore, crop improvement claims should be viewed as a future opportunity rather than a demonstrated application. To bridge this gap, future research should test condensate markers, scaffold proteins, RNA-binding proteins, chaperone-rich assemblies, and condensate-associated RNA cargos directly in crop tissues under field-relevant stress combinations. Priority systems should include heat–drought stress during flowering and grain filling, flooding–reoxygenation, salinity–heat stress, recurrent drought, and abiotic stress combined with pathogen pressure. Reviews from 2025 emphasize that understanding how condensates regulate signaling, RNA metabolism, and stress adaptation could support agricultural molecular breeding and crop-resilience strategies [146]. Parallel reviews in plant immunity likewise suggest that integrating condensate biology into plant defense research may open new routes for enhancing resilience and sustainability [147,148].

More generally, current abiotic-stress literature continues to stress the urgent need for stress-resilient crops under increasingly unpredictable environmental combinations. Thus, translational optimism should remain cautious [149]. Manipulating condensate behavior may produce beneficial effects on stress tolerance, but it could also disturb growth, development, or response specificity, especially because phase separation is deeply integrated with normal cellular organization [150]. The most realistic near-term goal is therefore not direct large-scale engineering, but identification of tractable regulatory nodes, such as proteins with stress-dependent condensation behavior, post-translational modifiers, or RNA-binding factors whose phase behavior can be tuned without severe pleiotropic cost [151]. From an agricultural perspective, these regulatory nodes should be connected with practical methods that directly address crop-production problems. A realistic translational pipeline would begin with controlled-environment screening of condensate markers under single and combined stresses, followed by validation in diverse cultivars and field-like stress regimes. Candidate condensate-associated genes or proteins could then be integrated with marker-assisted selection, genomic selection, genome editing, or stress-priming strategies. In parallel, nanosensors, portable biosensors, chlorophyll fluorescence imaging, thermal imaging, and high-throughput phenotyping can be used to monitor early stress signals before yield loss becomes visible. This combined strategy would allow condensate biology to move from cellular observation toward applied crop management, including early stress diagnosis, selection of resilient germplasm, and development of cultivars with improved recovery after recurrent heat, drought, salinity, or pathogen-associated stress. In this sense, condensate biology should be viewed as a possible future layer within broader stress-smart breeding, phenotyping, and biotechnology pipelines rather than as a stand-alone or already validated crop-improvement solution.

## 8. Conclusions

Biomolecular condensates provide a useful conceptual framework for understanding how plant cells may organize RNA fate, protein sequestration, signaling, transcriptional regulation, and recovery under stressful environments. Current evidence supports their roles as rapid stress-responsive assemblies, while their direct involvement in combined-stress prioritization and stress memory remains a testable hypothesis. The most important next step is to move from descriptive observation toward mechanistic validation by tracking condensate composition, material properties, assembly–disassembly kinetics, and functional outcomes across single, combined, sequential, and recurrent stress regimes. Such studies will clarify whether condensates are causal regulators of plant stress tolerance or markers of cellular stress state. With careful validation in crop systems, condensate biology may become a useful layer in future strategies for stress-resilient agriculture.

## Figures and Tables

**Table 1:** Physicochemical and molecular determinants of biomolecular condensate formation and dynamics in plant cells.

Factor Category	Key Determinants	Mechanism/Effect on Condensates	Relevance to Stress
Intrinsically disordered regions (IDRs)	Low-complexity sequences, prion-like domains	Promote multivalent weak interactions driving phase separation	Enable rapid condensate assembly under stress [82]
RNA abundance	mRNA concentration, RNA species diversity	Acts as scaffold or buffer for condensate formation	Alters SG/PB dynamics during stress [45,83]
Protein concentration	Local enrichment, stoichiometry	Threshold-dependent nucleation and growth	Stress-induced protein accumulation promotes condensates [84]
Post-translational modifications	Phosphorylation, ubiquitination, SUMOylation	Modulate interaction strength and phase behavior	Fine-tunes condensate assembly/disassembly [85]
Redox state	ROS levels, oxidative modifications	Alters protein interactions and material properties	Links oxidative stress to condensate remodeling [86,87]
pH	Cytoplasmic/nuclear pH shifts	Affects protein charge and solubility	Stress-induced pH changes regulate phase separation [88]
Ionic strength	Salt concentration, ion composition	Screens electrostatic interactions	Salinity stress directly impacts condensate stability [89,90]
Temperature	Heat stress	Enhances molecular mobility and phase transitions	Promotes stress granule formation [91,92]
Ca^2+^ signaling	Calcium fluxes	Influences protein interactions and signaling hubs	Integrates signaling with condensate behavior [93,94]

**Table 2:** Types of plant stress memory and hypothesized links to biomolecular condensate dynamics.

Memory Type	Description	Molecular Basis	Hypothesized Condensate Link	Example Outcome
Short-term memory	Transient enhanced response after stress	Protein modifications, signaling persistence	Temporary condensate assembly or altered dynamics	Faster response upon repeated stress [110,116]
Transcriptional memory	Sustained changes in gene expression	Chromatin modifications, transcriptional priming	Nuclear condensates regulating transcription hubs	Enhanced gene activation [117]
Post-transcriptional memory	Changes in mRNA stability or translation	RNA storage, decay control	SGs and PBs modulating RNA fate	Selective translation upon re-stress [118,119]
Proteostasis-based memory	Altered protein stability or localization	Protein aggregation, degradation pathways	Protein sequestration in condensates	Rapid protein reuse or degradation [120]
Metabolic memory	Persistent metabolic adjustments	Metabolite accumulation, enzyme regulation	Indirect via condensate-regulated enzymes	Improved stress tolerance [121]
Cross-stress memory	One stress influences response to another	Network rewiring, signaling crosstalk	Condensate remodeling alters selectivity	Altered response to different stress [27,122]
Epigenetic memory	Long-term heritable changes	DNA methylation, histone modification	Indirect link via nuclear condensates	Long-lasting adaptation [123]

**Table 3:** Technology–problem matching framework for studying biomolecular condensates in plant complex stress responses.

Technology/Approach	Main Problem Addressed	Plant Condensate Biology Insights	Agricultural or Translational Relevance
Live-cell confocal imaging	Dynamic observation of condensate formation and disappearance	Real-time nucleation, fusion, dissolution, subcellular movement, and recovery behavior under heat, drought, salinity, or combined stress	Identifies stress-responsive condensate markers that may indicate early cellular stress before visible injury
Super-resolution microscopy	Limited resolution of small or closely packed condensates	Fine spatial organization, nanoscale condensate architecture, interaction with organelles, and SG/PB proximity	Helps detect subtle cellular stress responses useful for screening tolerant genotypes
FRAP and related fluorescence recovery assays	Need to quantify condensate material properties	Molecular exchange rate, liquidity, gel-like behavior, residence time, and stress-induced hardening or reversibility	Distinguishes adaptive reversible condensates from potentially harmful persistent aggregates
Crosslinking-mass spectrometry	Weak and transient protein interaction networks are difficult to capture	Protein–protein and protein–RNA interaction networks within condensates under single versus combined stress	Identifies regulatory proteins or interaction hubs that can be targeted in breeding or biotechnology
Proximity labeling/AP-MS proteomics	Condensate composition is context-dependent	Stress-specific and shared condensate clients, scaffold proteins, chaperones, RNA-binding proteins, and signaling factors	Reveals candidate biomarkers for heat, drought, salinity, or pathogen-resilient crops
Single-cell transcriptomics	Tissue-level data hide cellular heterogeneity	Cell-type-specific transcriptional states associated with condensate-regulated stress responses	Helps identify which root, leaf, guard-cell, or vascular-cell populations contribute most to stress tolerance
Spatial transcriptomics	Loss of tissue-position information	Spatial organization of stress-responsive transcripts in relation to tissues and organelles	Connects condensate-linked regulation with tissue-level stress injury and recovery zones
RNA interactome capture/RIP-seq/CLIP-seq	Unknown RNA cargo of SGs, PBs, and nuclear condensates	Stress-regulated RNAs stored, repressed, degraded, or released by condensates	Identifies stress-memory transcripts and mRNA targets for improving recovery after stress
Genomics and mutant/overexpression analysis	Need to test causality rather than correlation	Functional importance of scaffold proteins, RNA-binding proteins, chaperones, and PTM regulators	Validates genes that may be used in marker-assisted selection, genome editing, or transgenic approaches
Nanosensors and field-compatible biosensors	Difficulty linking cellular condensates with real crop stress diagnosis	Real-time detection of ROS, Ca^2+^, pH, hormones, metabolites, or nutrient status in living tissues	Provides early-warning tools for drought, heat, salinity, and nutrient stress management in crops
Field phenotyping and controlled-environment stress platforms	Laboratory findings may not translate to agriculture	Links condensate traits with growth, yield, survival, and recovery under realistic stress combinations	Converts condensate biology into practical crop-screening and stress-management pipelines

## Data Availability

No new data were created or analyzed in this study. Data sharing is not applicable to this article.
